# Large novel deletions detected in Chinese families with aniridia: correlation between genotype and phenotype

**Published:** 2011-02-19

**Authors:** Xiaohui Zhang, Qingsheng Zhang, Yi Tong, Hanjun Dai, Xin Zhao, Fengge Bai, Liang Xu, Yang Li

**Affiliations:** 1 Beijing Institute of Ophthalmology, Beijing Tongren Eye Center, Beijing Tongren Hospital, Capital Medical University, Beijing Ophthalmology & Visual Sciences Key Laboratory, Beijing, China; 2 Puyang Eye Hospital, Henan, China; 3 The First Affiliated Hospital, Fujian Medical University, Fuzhou, Fujian, China

## Abstract

**Purpose:**

To describe the clinical and genetic findings in two Chinese families with aniridia and other ocular abnormalities.

**Methods:**

Two unrelated families were examined clinically. After informed consent was obtained, genomic DNA was extracted from the venous blood of all participants. Mutation screening of all exons of the *PAX6* (paired box gene 6) gene was performed by direct sequencing of PCR-amplified DNA fragments. Multiplex ligation-dependent probe amplification (MLPA) was performed to detect large deletions. Linkage analysis was used to validate the large deletions revealed by MLPA in all available family members.

**Results:**

Clinical examination and pedigree analysis revealed one four-generation family (85) and one three- generation family (86) with total aniridia, congenital cataracts, foveal hypoplasia, and glaucoma. No mutation in *PAX6* was identified after PCR-sequencing. Through MLPA analysis, a large deletion including the whole *PAX6* gene, *DKFZp686k1684* (hypothetical LOC440034), and the *RCN1* (reticulocalbin 1) gene was detected in family 85; a 3′ deletion to the *PAX6* gene including the *ELP4* (elongator complex protein 4) and the *DCDC1* (doublecortin domain containing 1) gene was identified in family 86.The two large deletions were confirmed with linkage analysis and the “loss of heterozygous” in the different *PAX6* regions were co-segregated with the phenotype of the two families, respectively.

**Conclusions:**

Patients with the *PAX6* contiguous gene deletion, including the *RCN1* gene, presented more severe vision impairments than those carrying the *PAX6* 3′ deletion. Large deletions may account for several Chinese families and sporadic cases with aniridia and screening for these kinds of alterations should be included in aniridia patients’ analyses.

## Introduction

Aniridia (AN; OMIM 106210) is a rare congenital disorder characterized by the complete or partial absence of the iris. The incidence of AN in the general population is about 1 in 64,000 to 96,000 [1]. Vision is usually impaired by other ocular abnormalities such as corneal opacification, cataract, glaucoma, fovea and optic nerve hypoplasia, and nystagmus [1]. About two-thirds of AN cases are families with an autosomal dominant mode of inheritance. In the remaining third no family history is found.

The aniridia gene was first mapped on chromosome 11p13 by linkage analysis, and then isolated by positional cloning in 1991 [2]. The *PAX6* (paired box gene 6) gene spans 22 kilobases and contains 14 exons, including an alternatively splicing exon5a. Therefore, there are two isoforms: PAX6 (−5a), comprising 422 amino acids, and PAX6 (+5a), comprising 436 amino acids [2,3]. *PAX6* encodes a transcription factor that is involved in several development pathways and is expressed early in the development of the eye, numerous regions of the brain, and the pancreas. PAX6 contains an NH_2_-terminal paired domain, a homeodomain separated by a glycine-rich linker sequence, and a COOH-terminal proline-serine-threonine rich transregulatory domain [2,3]. Most aniridia cases are caused by intragenic mutations of *PAX6*, which include nonsense mutations, splicing mutations, frame-shifting insertions or deletions, in-frame insertions or deletions, missense mutations, and run-on mutations [2-7]. A small numbers of aniridia cases can be due to large chromosomal deletions or rearrangements [2,8]. Aniridia generally occurs in isolation or accompanied by other ocular malformations, but it occurs, more rarely, as part of the WAGR (Wilms’ tumor, aniridia, genitourinary abnormalities, and mental retardation) syndrome (OMIM 194072) [9]. WAGR is usually caused by deletions of chromosome 11p13, which include *PAX6* and *WT1* (Wilms tumor 1) [9]. As large deletions could not be identified by the routine PCR-sequencing mutation detection method, only a few isolated aniridia patients with the large deletions in the *PAX6* region have been documented and the most of them are sporadic cases [2,8-18].

In this study, we describe the clinical findings in two Chinese families with two different large deletions in the region of *PAX6*.

## Methods

### Patients and DNA sample collection

This study was performed according to the tenets of the Declaration of Helsinki for research involving human subjects. This study was approved by the Beijing Tongren Hospital Joint Committee on Clinical Investigation, Beijing, China. After informed consents were obtained, participants underwent ophthalmologic examination including bilateral best corrected visual acuity using E decimal charts, slit-lamp biomicroscopy inspection of the anterior chamber, intraocular pressure (IOP) measurement by applanation tonometry (Goldmann), and fundus examination with a 66-diopter VOLK lens. Some patients underwent electroretinography (ERG) and A/B ultrasonic scan examination.

### Mutation screening of *PAX6*

Peripheral blood was obtained by venipuncture and genomic DNA was extracted according to standard protocols. The 14 exons of *PAX6* were amplified by polymerase chain reaction (PCR) from genomic DNA. Thirteen pairs of primers for *PAX6* were used (Table 1), according to the article previously published [17]. For direct sequencing, PCR products were purified (Shenneng Bocai PCR purification kit; Shenneng, Shanghai, China). An automatic fluorescence DNA sequencer (ABI, Prism 373A; Perkin Elmer, Foster City, CA), used according to the manufacturer’s instructions, sequenced the purified PCR products in both forward and reverse directions. DNAssit, version 1.0 compared nucleotide sequences with the published DNA sequence of *PAX6* (GenBank NM_001604.3).

**Table 1:** *PAX6* PCR primers used in this study

**Primer**	**Forward (5'-3')**	**Reverse (5'-3')**	**Tm (°C)**	**Product size (bp)**
exon1	AGGGAACCGTGGCTCGGC	GGGTGAGGGAAGTGGCTGC	62	207
exon2	TTATCTCTCACTCTCCAGCC	GGAGACCTGTCTGAATATTGC	54	307
exon3	TCAGAGAGCCCATGGACGTAT	CTGTTTGTGGGTTTTGAGCC	58	193
exon4	AGTTCAGGCCTACCTGATGC	GTCGCGAGTCCCTGTGTC	58	201
exon5	CTCCCTCATCTTCCTCTTCC	GGGGTCCATAATTAGCATCG	58	327
exon6-7	GGGCTACAAATGTAATTTTAAGAAA	AGAGAGGGTGGGAGGAGGTA	56	509
exon8	GAGCTGAGATGGGTGACTG	GAGAGTAGGGGACAGGCAAA	58	300
exon9	AGACTACACCAGGCCCCTTT	TGAAGATGTGGCATTTACTTTGA	58	291
exon10	GGAACCAGTTTGATGCACAG	ACTCTGTACAAGCACCTCTGTCTC	58	243
exon11	GGGCTCGACGTAGACACAGT	GGAAACTGAGGGCAAGAGAA	56	300
exon12	CGGGCTCTGACTCTCACTCT	GCCACTCCTCACTTCTCTGG	60	220
exon13	GCTGTGGCTGTGTGATGTGT	AGGAGATTCTGTTTGGGTA	52	281
exon14	TCCATGTCTGTTTCTCAAAGG	TCAACTGTTGTGTCCCCATAG	56	219

### Multiplex ligation-dependent probe amplification (MLPA analysis)

MLPA was performed with SALSA MLPA Kits P219 (Amsterdam, the Netherlands) according to the manufacturer’s instructions. In brief, 100 ng DNA was denatured and hybridized with the SALSA probe mix overnight at 60 °C. The samples with ligase 65 were incubated for 15 min at 54 °C, after which PCR amplification was performed with the specific SALSA FAM PCR primers. The PCR products were separated by capillary electrophoresis on an automatic fluorescence DNA sequencer (ABI, Prism 373A; Perkin Elmer). Data analysis was performed by exporting the peak areas to a Microsoft Excel (Microsoft Corporation, Redmond, WA) file. Each peak was first normalized as described elsewhere [17] and the normalized peak was then divided by the mean of that peak in the control samples. The ratios outside the range of 0.7–1.3 times the control peak area were considered abnormal, with those below 0.7 representing deletions and those above 1.3 representing duplications. For each MLPA analysis, several normal controls were included and the standard deviation for the normal samples was usually less than 10% of the mean. Each result was confirmed by two independent tests.

### Linkage analysis

**Figure 1:**
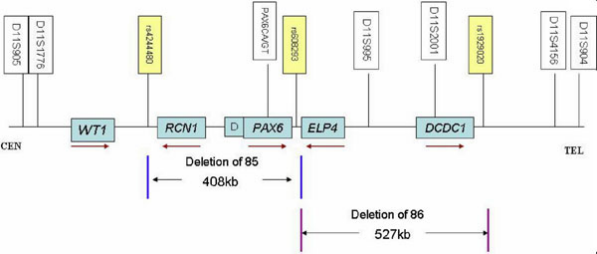
Schematic representation of *WT1/ RCN1/ DKFZ p686k1684/PAX6/ ELP4/ DCDC1* on chromosome 11p13 and the relative positions of the microsatellite markers and single nucleotide polymorphism (SNP) used in linkage and real-time quantitative PCR analysis. D presents *DKFZ p686k1684*. Brown arrows below the each gene indicated the transcription direction for each gene. The region between the two blue vertical lines presents the deleted region (407 Kb) of family 85, the region between the two purple vertical lines presents the deleted region (527 Kb) of family 86.

To validate the large deletions detected by MLPA, genotyping for families 85 and 86 was performed with the following 8 microsatellite markers: D11S905, D11S1776, GDB.250586, PAX6.CA/GT, D11S995, D11S2001, D11S4156, and D11S904. The fine mapping primer sequences were obtained from the GDB (Human Genome Database). The positions of these markers related to *WT1*, *RCN1* (reticulocalbin 1), *DKFZ p686k1684* (hypothetical LOC440034),* PAX6, ELP4* (elongator complex protein 4)*,* and *DCDC1* (doublecortin domain containing 1) are shown in Figure 1.

### Real-Time Quantitative PCR analysis

To define the relative exact break point of the deletions, real-time quantitative PCR was performed in the affected members of the two families round the three regions (3′ to *RCN1*, 3′ to *PAX6*, and near the D11S4156 locus). Real-time quantitative PCR reactions were performed on the Rotor-Gene 6000 (Corbett Research, Mortlake, NSW, Australia) in a final volume of 10 μl, containing 300 nM primers and 1 μl (100 ng) genomic DNA, using the Eva green PCR Master Mix (Bio-Rad Laboratories, Hercules, CA). The primers in the analysis are shown in Table 2. Each assay was done in triplicate. The relative quantitation (RQ) of target gene was accomplished using RQ manager software (Bio Rad systems) and was calculated using the 2^-ddCt^ method [19]. All experimental samples were normalized using human *GAPDH* as an internal control. The significance of the difference with a reference experiment was calculated with Student’s *t*-test.

**Table 2:** Primers sequences and the physical position used in real-time quantitative PCR and the results of MLPA and real-time PCR.

			**Family 85**		**Family 86**	
**Gene/SNP**	**Physical position**	**Primer sequences (5′-3′)**	**MLPA**	RQ result*	**MLPA**	RQ result*
rs11031332	31235538	Fwd: CATTAGATTCATGTGGCTTGTTG				1.17
		Rev: AATCCGATTCCTCGATTTGT				
rs1929020	31237204	Fwd: TGACTGGTGCTACTGCCAAC				0.49
		Rev: CTCCCGAATAGCGTGGATTA				
*DCDC1*	31240747–31347897		-		+	
*ELP4*	31487873–31761905		-		+	
rs35674517	31763272–31763273	Fwd: TTCTTCCCCATTTTCTTTTCAA		1.18		
		Rev: TTCAGAATGTTCATAATGCTTTCAA				
rs608293	31764856	Fwd: GAATATTTTTCCCCCAAAGC		0.44		1.07
		Rev: TGAGAGGCCCAGAGTAAAAAGA				
*PAX6*	31762916–31796085		+		-	
*DKFZp686K1684*	31794690–31865163		+		-	
*RCN1*	32074656–32083376		+		-	
SHGC-143238	32102437–32102880	Fwd: TCCTCCCCTTGAGTCACTTACAT		0.5		
		Rev: TTACTTGCCAAAACACACTCCCT				
rs7104670	32155485	Fwd: GTTACCACATTCGCAAAGCA		0.46		
		Rev: CAGACAAACACATTGACAGTCCT				
rs12577592	32165037	Fwd: CCTTGCTCCTTTCAGTCCAG		0.35		
		Rev: ACCCTGAGTGTGTGTCATGC				
rs4244480	32172819	Fwd: ACTTATCCCGCCCTTGTGTT		0.41		
		Rev: CACCAGGTTGTTTTGGGACT				
rs72320219	32173790–32173791	Fwd: AGGAAAATCGCTTGAACCTG		1.08		
		Rev: GGGGTGGTTGTGAGGACTAA				
*GAPDH*	6516714–6517797	Fwd: ACCCAGAAGACTGTGGATGG				
		Rev: TTCTAGACGGCAGGTCAGGT				

Abbreviations: MLPA, Multiplex ligation-dependent probe amplification; RQ, relative quantity; +, deletion ; -, no deletion; *mean value of three times; Fwd, forward; Rev, reverse.

## Results

### Clinical findings

**Figure 2:**
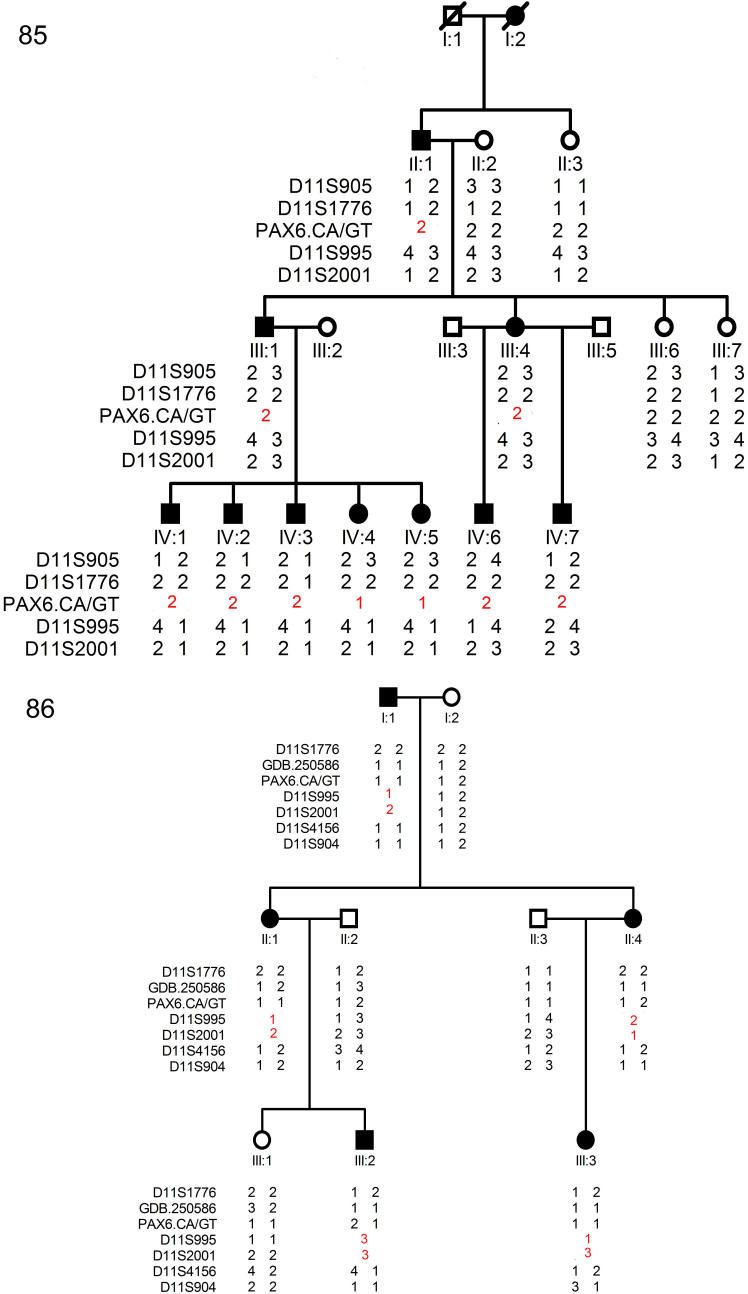
Family structure and haplotype analysis of two Chinese families with aniridia. Pedigree and haplotype analysis of family 85 and 86 with aniridia showed “loss of heterozygous” segregation with the microsatellite marker PAX6.CA/GT (family 85), D11S995, and D11S2001 (family 86), respectively. All markers are on chromosome 11, listed in descending order from the centromeric end. Squares indicate males; circles indicate females; slashed symbols indicate deceased; solid symbols indicate affected; open symbols indicate unaffected.

**Figure 3:**
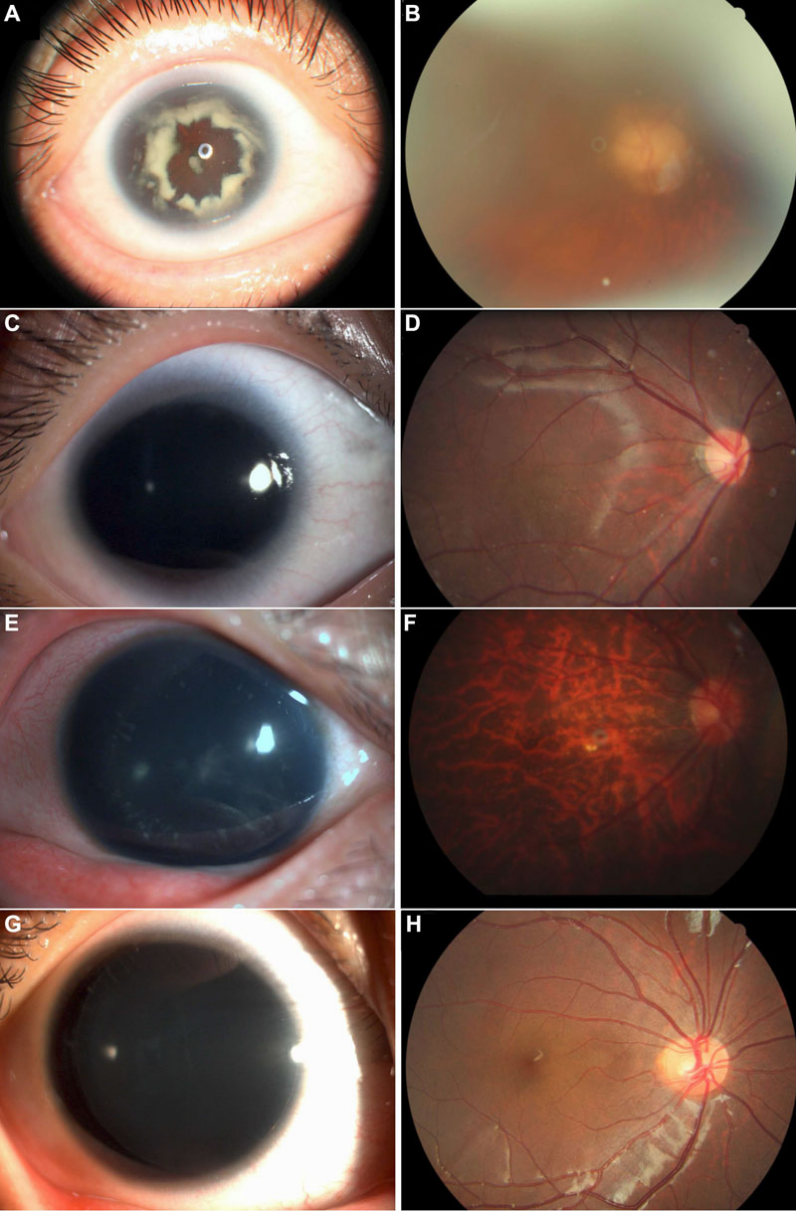
Ophthalmological findings in patients from the two families. **A**: Photograph of anterior segment of patient III-4 of 85 with complete absence of iris and the progressing dense congenital cataract. **B**: Fundus images of patient III-4 showed late-stage glaucomatous cupping of the optic disc. **C**: Complete hypoplasia of the iris and congenital cataract were observed in patient IV-7 of family 85. **D**: Fundus images of patient IV-7 showing foveal hypoplasia. **E**: Photograph of anterior segment of patient II-4 of family 86 with complete absence of iris and congenital cataract. **F**: Fundus image of patient II-4 showing a tessellated appearance. **G**: Photograph of the anterior segment of patient III-3 of family 86 with complete absence of iris and mild cataract. **H**: Fundus image of patient III-3 showing a normal foveal reflex.

We have identified one four-generation family (#85) and one three- generation family (#86) with aniridia. The inheritance pattern in the families was autosomal dominant (Figure 2). After clinical examinations and a review of hospital records, 11 individuals in family 85 were found to have aniridia. All patients presented bilateral complete absence of iris, severe congenital nystagmus, and congenital cataracts (Figure 3A,C). Foveal hypoplasia was observed in all fourth-generation patients except (IV-6; Figure 3D). The ERG of patient IV-7 showed slight cone cell dysfunction. The proband (III-4), her father, and her brother presented high intraocular pressure (IOP) and late stage glaucoma changes in the optic disc (Figure 3B). Due to the progressive density of the lens opacification, the fovea of patients in the second and third- generation and patient IV-6 could not be observed clearly. In family 86, five patients were identified and all patients had bilateral complete absence of iris and congenital cataracts (Figure 3E,G). Neither mental retardation nor other general abnormalities was observed or documented in all patients from the two families. Their detailed clinical features are summarized in Table 3.

**Table 3:** The clinical features of patients in the two Chinese families.

**Family number**	**Patient number**	**Gender/Age year**	**Best corrected vision (OD/OS)**	**IOP**	**Nystagmus**	**Aniridia**	**Congenital cataract**	**Glaucoma**	**Foveal hypoplasia**
85	II-1	M/64	NLP	NA	S	C	YES	Late stage	NA
	III-1	M/43	NLP	NA	S	C	YES	Late stage	NA
	III-4	F/39	0.02/0.02	28/25	S	C	YES	Late stage	NA
	IV-1	F/11	0.05/0.05	NA	S	C	YES	NO	YES
	IV-2	M/7	NA	NA	S	C	YES	NO	YES
	IV-3	M/6	NA	NA	S	C	YES	NO	YES
	IV-4	M/5	NA	NA	S	C	YES	NO	YES
	IV-5	M/3	NA	NA	S	C	YES	NO	NA
	IV-6	M/18	0.1/0.1	NA	S	C	YES	NO	NA
	IV-7	M/7	0.1/0.1	17/25	S	C	YES	NO	YES
86	I-1	M/71	0.2/0.4	NA	NO	C	YES	NO	NO
	II-1	F/39	0.4/0.4	13/14	NO	C	YES	NO	NO
	II-4	F/37	0.2/0.02*	16/11	NO	C	YES	NO	NO
	III-2	F/9	0.4/0.6	19/18	NO	C	YES	NO	NO
	III-3	M/7	0.8/0.6	10/12	NO	C	YES	NO	NO

Abbreviations: M, male; F, female; BCVA, best-correct visual acuity; OD, right eye; OS, left eye; NLP, no light peception; IOP, introocular pressure; NA, unavailable; S, sever; C, complete; *cornea of left eye was opacified due to the complication of implantation of iris diaphragm intraocular lens.

### Mutation analysis

By the direct sequencing of 14 exons of *PAX6*, no mutation was detected in the two families.

### MLPA Results

**Figure 4:**
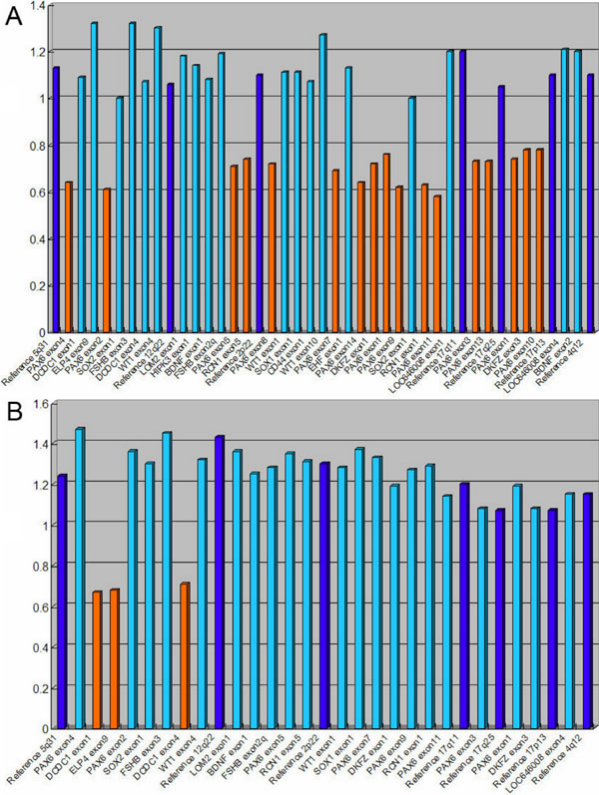
The normalized MLPA results of the probands of the two families. **A**: The normalized MLPA result of III-4 of family 85. **B**: The result of II-4 of family 86. The height of the columns represents of the dosage of the respective segments in the genomic DNA with two alleles. The light blue columns represent chromosome 11p13 specific probes. The orange columns represent the deleted probes. The dark blue columns represent the control probes. The allele dosage of the deleted probes was found in the range of about 0.5–0.7 of normal control, which corresponds to one allele.

Using the MLPA Kits P219, two different deletions were detected in the two families (Figure 4). In family 85, a deletion of the whole *PAX6* gene, the *DKFZ p686k1684* gene, and the *RCN1* gene was found; in family 86, a deletion of the *ELP4* gene and the *DCDC1* gene, which is located in the 3′ region of the *PAX6* gene, was identified.

### Genotyping Results

The two families were genotyped with several STRP markers located around *PAX6* in the chromosome 11p13 region. The linkage analysis results were highly informative for the two families. For family 85, all patients display “loss of heterozygosis” at marker PAX6 CA/GT, which is located inside the *PAX6* gene. All patients in family 86 show “loss of heterozygosis” at markers D11S995 and D11S2001, which is closed to *ELP4* and *DCDC1* (Figure 2).

### Real-Time Quantitative PCR analysis results

**Figure 5:**
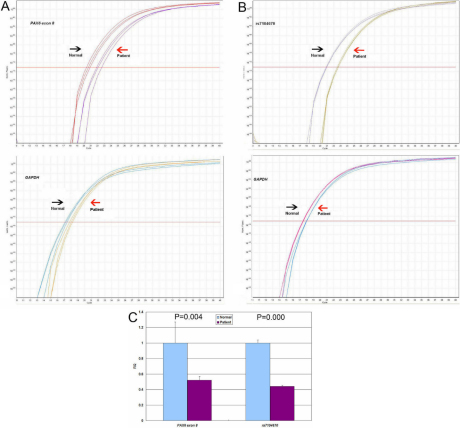
Analysis of deletions in the *PAX6* gene region by real-time quantitative PCR. **A**: Fluorescence amplification plots of the real-time quantitative PCR for exon 8 of the *PAX6* gene, detected deletion by MLPA. **B**: Fluorescence amplification plots of the real-time PCR for rs7104670. Arrows in **A** and **B** indicated triplicate signals obtained for a control subject (Normal) and the patient III-1 of family 85 (Patient). **C**: Histogram indicated the relative quantity (RQ) between the exon8 of *PAX6* or rs7104670 and *GAPDH* values. Bars represent mean values±standard deviations for the triplicate values.

This study set up a real-time quantitative PCR assay to define the relative breakpoint of the deletions. *GAPDH* (glyceraldehyde-3-phosphate dehydrogenase) was used to normalize *PAX6* values. Assay for exon 8 of *PAX6* were set up by using the patients in family 85 with the *PAX6* deletion. The amplification plots are shown in Figure 5A,B, and the patient had about a half RQ value with respect to the normal control Figure 5C. Thus this study used this assay to analyze several single nucleotide polymorphisms (SNPs) around *RCN1*, *PAX6*, *ELP4*, and *DCN1*. The results were summarized in Table2. The relative exact breakpoints for the two families were showed in Figure 1.

## Discussion

In this study, we described two Chinese families with aniridia and other ocular abnormalities. Using MLPA, a large deletion, including *PAX6*, *DKFZ p686k1684*, and *RCN1*, was identified in family 85; a deletion of *ELP4* and *DCDC1*, leaving *PAX6* intact, was found in family 86. The two deletions were co- segregated with the phenotype in the families, respectively.

Until now, almost 300 intragenic mutations of *PAX6* have been documented in the *PAX6* allelic variation database [2-7]. Most of the intragenic mutations lead to premature protein truncation, which is likely to be acted on by nonsense-mediated decay (NMD). By reviewing the literature on genotype-phenotype correlation studies, the mutations that introduce premature terminated codons (PTCs) are consistently associated with aniridia or closely related phenotypes [6,7]. The patient carrying the complete deletion of *PAX6*, observed by Vincent et al. [15], did not present distinctive or more severe clinical manifestations than those associated with nonsense mutations. However, more severe bilateral visual impairment was observed in all the patients of family 85. The proband’s father and brother totally lost their sight at the age of 40 due to glaucoma. Several patients showed foveal hypoplasia. The large deletion detected in family 85 was novel and contained not only the complete *PAX6* gene but also *DKFZ p686k1684* and *RCN1*, which are located about 300 kb upstream of *PAX6*. RCN1 (reticulocalbin 1), resident in the endoplasmic reticulum, is a Ca^2+^ binding protein that participates in the secretory pathway and is expressed in the eye [20].Linkage between *Pax6* and *Rcn1* has been conserved in mice, humans, and fish [21]. *Pax6* was originally isolated in the mouse and mutations in the gene are responsible for the *small eye* phenotypes. The mouse *small eye* phenotype had already been suggested through homology mapping to be the mouse counterpart of human aniridia [22]. Recently, Favel et al. [21] observed that the mouse, carrying a heterozygous *Pax6* and *Rcn1* contiguous deletion, presented an extreme microphthalmia phenotype. They inferred that *Rcn1* might directly or indirectly contribute to the eye phenotype in *Pax6* contiguous gene deletions. The severe visual impairment observed in family 85 seemed to be consistent with the phenotypes found in the mouse described by Favel et al. [21]. *DKFZ p686k1684*, located between *PAX6* and *RCN1*, is a non-coding RNA with its function unclear[23].

The 3′ deletion identified in family 86 contained *ELP4* and *DCD4*, which are located downstream of *PAX6*. The deletions in this region, which contains 3′ regulatory elements for *PAX6*, were documented in several earlier studies [12-18]. Most patients harboring the 3′ deletions had only aniridia and other ocular abnormalities, which is similar to the phenotype observed in most nonsense mutations patients. The patients in family 86 showed mild vision impairments due to aniridia and congenital cataracts. Davis et al. [18] described a patient carrying a 1.3 Mb deletion, including several additional genes expressed in the brain, who also presented with autism and mental retardation.

In this study, the aniridia in both families was caused by large deletions in the *PAX6* region. In general, patients with *PAX6* contiguous deletion, including *RCN1,* may have relatively severe phenotypes. Large deletions may account for several Chinese families and sporadic cases with aniridia and screening for these kinds of alterations should be included in the aniridia patient’s analysis.
